# Cerebral Venous Sinus Thrombosis Presenting as an Acute Stroke Syndrome

**DOI:** 10.7759/cureus.101637

**Published:** 2026-01-15

**Authors:** Kevin Rivera, Nicholas Harvey, David Capote, Samuel Wagner

**Affiliations:** 1 Internal Medicine, Mount Carmel Health System, Columbus, USA; 2 Internal Medicine, Mount Carmel Health System, Grove City, USA

**Keywords:** acute stroke mimic, cerebral venous sinus thrombosis, headache, thrombophilia, transient ischemic attacks

## Abstract

Cerebral venous sinus thrombosis (CVST) is an uncommon cause of stroke and can present in ways that closely resemble arterial transient ischemic attack or ischemic stroke. Early recognition is important because management differs from typical stroke pathways, and initial noncontrast head CT may be normal.

We report a 63-year-old male patient with a history of remote pulmonary embolism, pulmonary hypertension, and a family history of venous thromboembolism who presented with several weeks of headache and neck pain followed by acute left-sided weakness. Initial computed tomography (CT) imaging showed no hemorrhage. CT venography revealed extensive dural venous sinus thrombosis involving the superior sagittal sinus, right transverse sinus, right sigmoid sinus, and extension into the distal right internal jugular vein, without arterial stenosis or large vessel occlusion. MRI with contrast confirmed near-complete involvement of the superior sagittal sinus with extension into the bilateral transverse sinuses. His neurologic deficits resolved quickly, thrombolysis was deferred, and anticoagulation was initiated with intravenous heparin followed by rivaroxaban. A comprehensive evaluation for inherited and acquired thrombophilia was negative.

This case highlights the need to consider CVST in patients with headache preceding focal neurologic deficits, even when the initial stroke evaluation is unrevealing. It also demonstrates that extensive clot burden may occur in the absence of an identifiable thrombophilic disorder. Anticoagulation remains the primary treatment, and escalation to endovascular therapy should be guided by clinical course and anatomic patterns rather than clot burden alone.

## Introduction

Cerebral venous sinus thrombosis (CVST) is an uncommon but clinically important cause of stroke, accounting for a small proportion of cerebrovascular events [1]. While it most often affects younger patients and women of reproductive age, CVST can occur across the adult lifespan and may present in ways that resemble an arterial ischemic stroke [1,2]. Despite advances in neuroimaging and earlier recognition, diagnosis remains challenging because presenting symptoms are heterogeneous and early imaging can be unrevealing [2,3].

The pathophysiology of CVST reflects Virchow’s triad of venous stasis, endothelial injury, and hypercoagulability [3,4]. In the International Study on Cerebral Vein and Dural Sinus Thrombosis (ISCVT), approximately one-third of patients had an identifiable inherited or acquired thrombophilia, though a substantial minority remained idiopathic after evaluation [5,6]. Headache is the most common presenting symptom and may occur in isolation early in the disease course, whereas focal neurologic deficits, seizures, or altered mental status are more typical in extensive disease [1,7]. Seizures at presentation are more frequently associated with frontal involvement or hemorrhagic venous infarction [8]. Older age, depressed consciousness, and focal motor deficits at presentation have been associated with worse outcomes [9], and involvement of the deep venous system carries a higher risk of disability or death [10]. Intracranial hemorrhage may be the initial radiographic finding in some patients [11]. Less common anatomic patterns, including extension into the internal jugular vein, have been described [12]. Dural arteriovenous fistulas may coexist with CVST or develop later, underscoring the importance of clinical awareness during follow-up [13,14].

Diagnosis relies on dedicated venous imaging. Magnetic resonance imaging (MRI) with magnetic resonance venography (MRV) is generally preferred, though computed tomography (CT) venography is a practical alternative in acute settings [1,2]. Anticoagulation remains the cornerstone of treatment, while endovascular approaches are reserved for select patients with clinical deterioration or failure to improve on anticoagulation alone [1-3,15]. Clinical-radiologic tools such as the Poor Response to Anticoagulation Therapy in Cerebral Venous Thrombosis (PRACT-CVT) score may help identify patients at higher risk of deterioration [16]. In selected cases involving the deep venous system, endovascular therapy has been associated with radiographic recanalization and functional improvement in case series, though risks remain significant [17]. We present a case of extensive multisinus CVST in an older male identified during a stroke evaluation, followed by a comprehensive but unrevealing thrombophilia workup.

## Case presentation

A 63-year-old White male patient with a history of remote pulmonary embolism not on anticoagulation, pulmonary hypertension, and a family history of venous thromboembolism presented with acute left-sided weakness and persistent headache.

Four weeks prior to presentation, he developed left-sided neck pain and intermittent headaches that began behind the left ear and radiated toward the temple. Over time, the headaches evolved into dull bifrontal pain lasting several hours and did not improve with nonsteroidal anti-inflammatory medications. He associated symptom onset with strenuous outdoor work and recalled a tick bite approximately seven weeks earlier without rash. Two weeks prior to admission, he experienced his most severe headache, accompanied by a single episode of nonbloody emesis; there was no history of dehydration. At that time, he sought evaluation at an urgent care clinic, where he was prescribed prednisone 50 mg orally once daily for five days, which was completed prior to his emergency department presentation. A noncontrast CT of the head was unremarkable. Lyme and Babesia testing were negative, and doxycycline was prescribed empirically. His neck pain was attributed to musculoskeletal strain, and he received a brief steroid taper and muscle relaxant without improvement.

Approximately one hour prior to arrival, he developed a sudden onset of left facial, arm, and leg weakness. He denied fever, chest pain, dyspnea, vomiting, diarrhea, arthralgias, or rash. On presentation, blood pressure was 153/92 mmHg, heart rate 90 beats per minute, respiratory rate 24 breaths per minute, oxygen saturation 100% on room air, and temperature 97.5°F. He appeared anxious but was alert and oriented. Cardiovascular and pulmonary examinations were normal. The abdomen was soft and nontender, and there was no peripheral edema. Neurologic examination demonstrated intact speech and comprehension. Cranial nerves II through XII were intact without dysarthria, facial asymmetry, or sensory loss. Strength was full except for a subtle reduction in left grip strength, corresponding to a National Institutes of Health Stroke Scale score of 1. There was no pronator drift.

Initial laboratory testing demonstrated polycythemia and mild thrombocytosis with otherwise normal renal function, hepatic function, and coagulation parameters (Table 1). The electrocardiogram showed sinus rhythm without ischemic changes.

**Table 1:** Initial laboratory results at presentation WBC: white blood cell; BUN: blood urea nitrogen; GFR: glomerular filtration rate; AST: aspartate aminotransferase; ALT: alanine aminotransferase; PT: prothrombin time; PTT: partial thromboplastin time; INR: international normalized ratio; TSH: thyroid-stimulating hormone; VBG: venous blood gas; pCO2: partial pressure of carbon dioxide; HCO3: bicarbonate

Test	Value	Reference range
WBC	9.0 K/µL	4.6-10.2 K/µL
Hemoglobin	17.6 g/dL	13.5-17.5 g/dL
Platelets	425 K/µL	142-424 K/µL
Sodium	137 mmol/L	136-145 mmol/L
Potassium	3.7 mmol/L	3.6-5.1 mmol/L
Glucose	129 mg/dL	70-99 mg/dL
BUN	13 mg/dL	8-20 mg/dL
Creatinine	1.03 mg/dL	0.60-1.30 mg/dL
GFR	82 mL/min/1.73 m²	>60 mL/min/1.73 m²
AST	12 U/L	15-41 U/L
ALT	16 U/L	7-52 U/L
Total bilirubin	0.7 mg/dL	0.3-1.2 mg/dL
Albumin	4.2 g/dL	3.5-4.8 g/dL
PT	13.2 sec	11.9-14.7 sec
PTT	29.1 sec	23.3-35.3 sec
INR	0.9	<5.0
Lactate	2.2 mmol/L	0.5-2.0 mmol/L
Troponin (hs)	7 ng/L	<20 ng/L
TSH	2.69 mIU/mL	0.45-5.33 mIU/mL
pH (VBG)	7.48	7.31-7.41
pCO2 (VBG)	34 mmHg	41-51 mmHg
HCO3 (VBG)	25.1 mmol/L	21-28 mmol/L

Imaging

**Figure 1:**
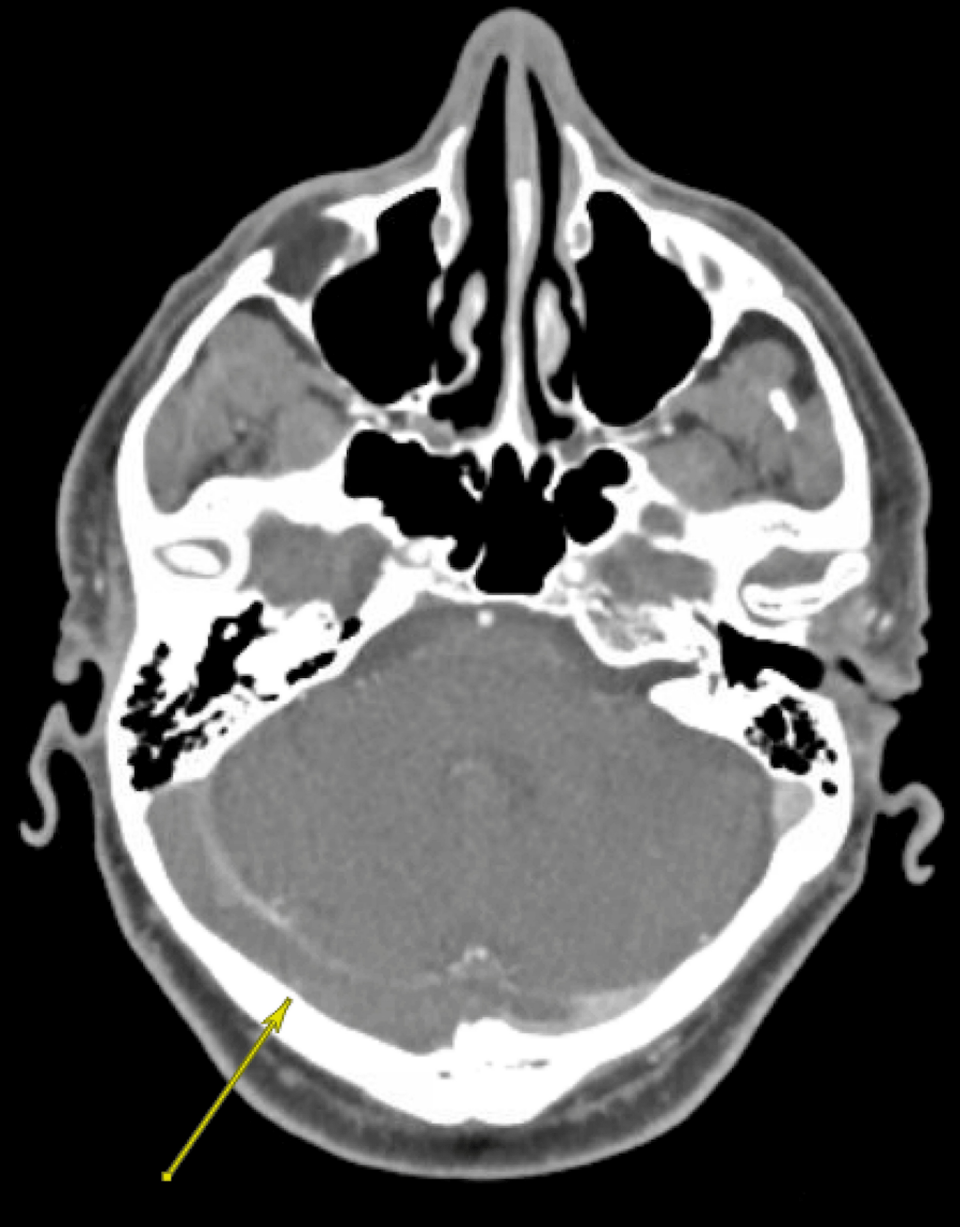
CT venography of the head, axial view, demonstrating filling defects within the right transverse sinus (arrow)

Noncontrast CT of the head demonstrated no acute intracranial hemorrhage, mass effect, or midline shift. CT venography of the head revealed extensive dural venous sinus thrombosis involving the superior sagittal sinus, right transverse sinus, and right sigmoid sinus, with extension into the distal right internal jugular vein (Figure 1). There was no evidence of arterial stenosis, dissection, or large vessel occlusion. CT perfusion imaging demonstrated no ischemic penumbra.

**Figure 2:**
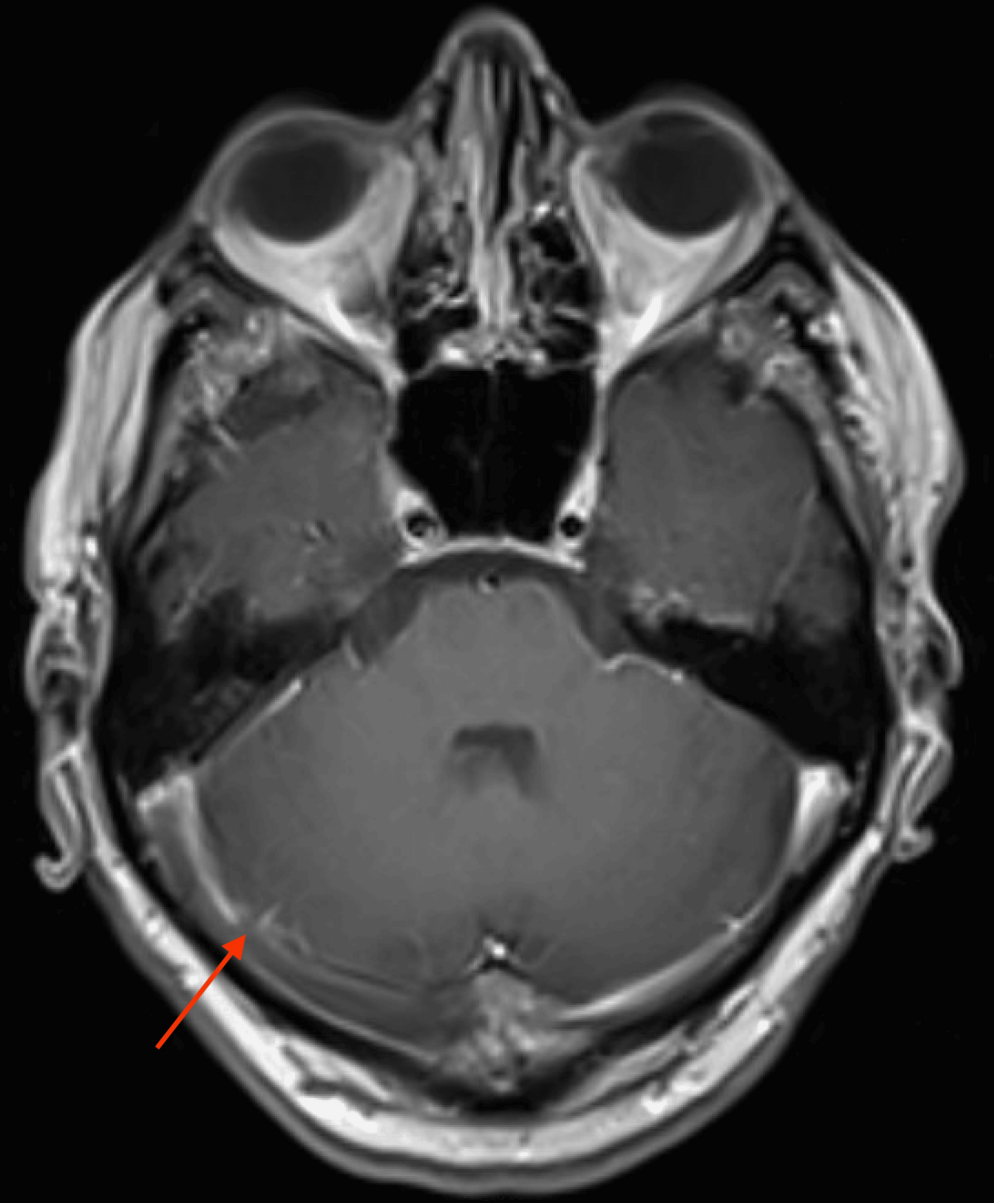
MRI of the brain with contrast, axial view, showing near-complete thrombosis of the superior sagittal sinus with extension into the right transverse sinus (arrow)

**Figure 3:**
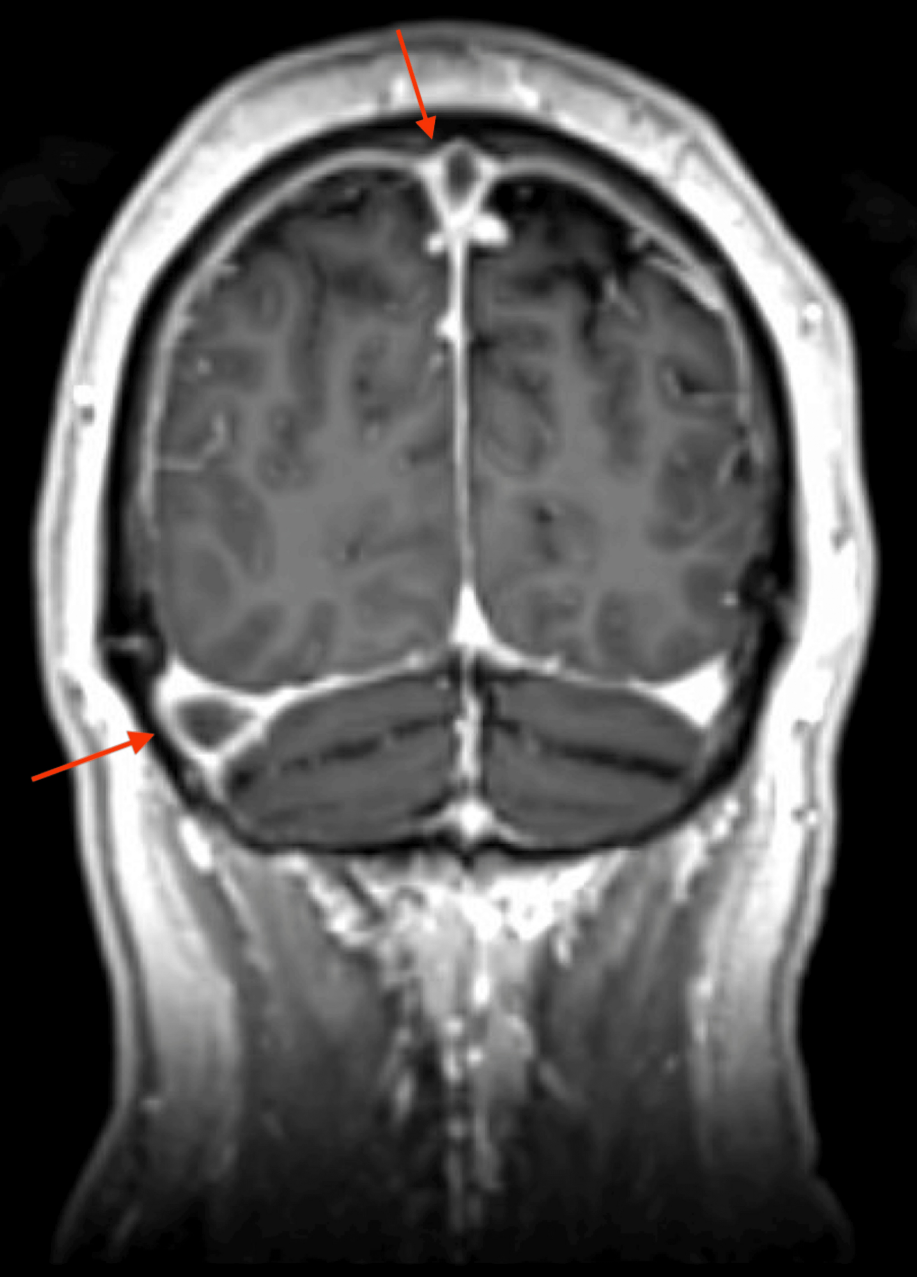
MRI of the brain with contrast, coronal view, demonstrating multisinus involvement including the right transverse sinus (arrow) and superior sagittal sinus (arrow)

**Figure 4:**
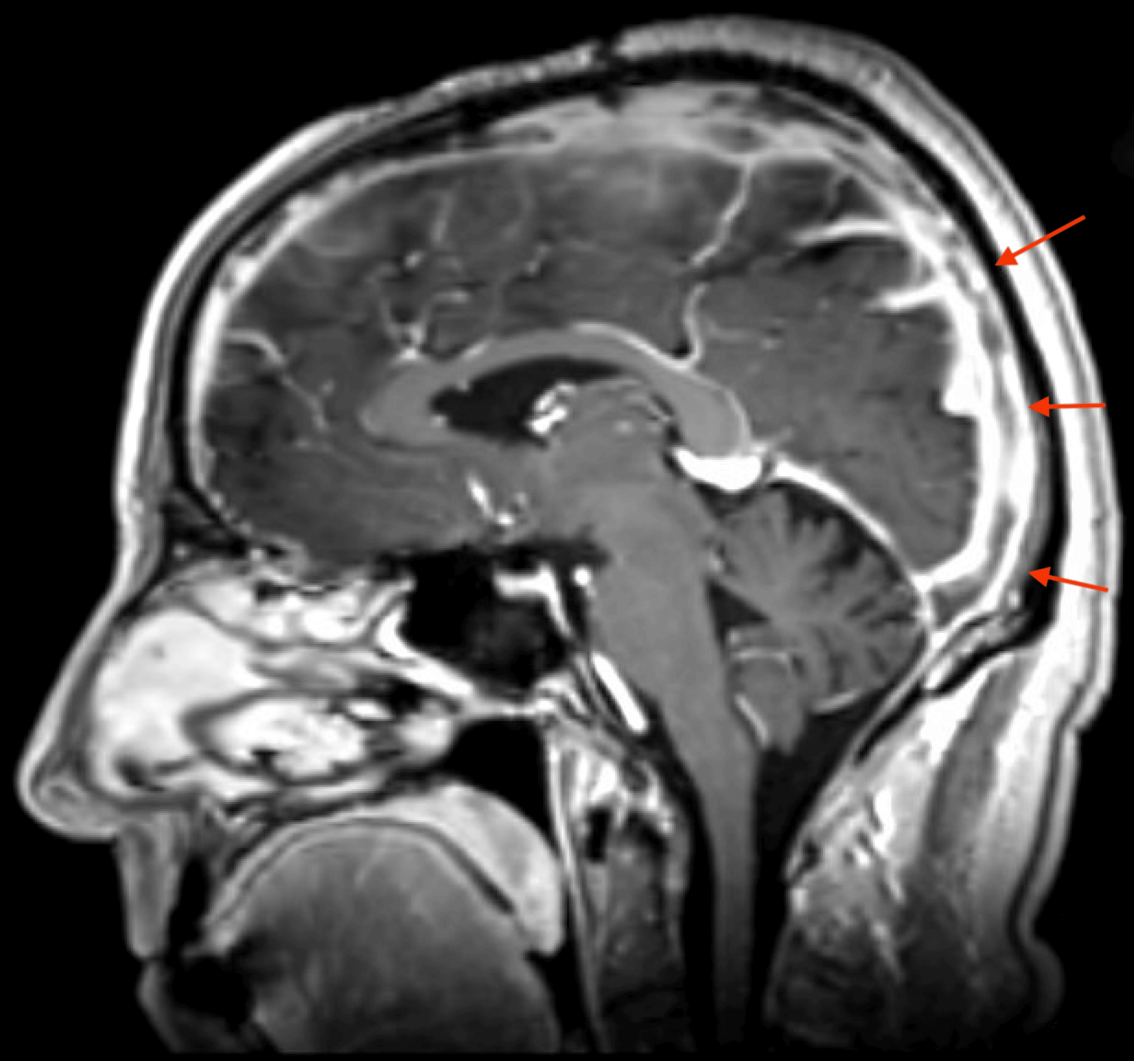
MRI of the brain with contrast, sagittal view, demonstrating multisinus involvement including the superior sagittal sinus (arrows)

MRI of the brain with contrast confirmed extensive venous sinus thrombosis with near-complete involvement of the superior sagittal sinus and extension into the right transverse and sigmoid sinuses, and partial involvement of the left transverse sinus (Figures 2-4). Major intracranial arteries were patent without evidence of aneurysm or occlusion.

Hospital course

Given the rapid improvement of neurologic deficits with resolution of weakness on reassessment, systemic thrombolysis was deferred. Intravenous unfractionated heparin was initiated using weight-based dosing and continued for two days. Neurology concluded that the transient weakness represented a transient ischemic attack in the setting of extensive dural venous sinus thrombosis. The patient was subsequently transitioned to rivaroxaban 15 mg orally twice daily for 21 days, followed by 20 mg orally once daily with plans for indefinite anticoagulation per hematology recommendations.

Transthoracic echocardiography demonstrated preserved left ventricular systolic function with an ejection fraction of 56%, grade I diastolic dysfunction, no intracardiac thrombus, and normal right ventricular systolic pressure. Lipid testing revealed hyperlipidemia, and hemoglobin A1c was 5.7%. He was discharged in stable condition.

A comprehensive thrombophilia evaluation returned several weeks later and was negative for inherited or acquired thrombophilia (Table 2). Repeat MRI or MR venography was not performed, as his neurologic symptoms resolved, and there was no clinical indication for repeat imaging.

**Table 2:** Hypercoagulable laboratory testing DRVTT: dilute Russell's viper venom time

Test	Result	Reference range
Protein C activity	124%	70-130%
Protein S activity	115%	65-140%
Antithrombin III activity	84%	80-120%
Cardiolipin IgA	3.4 APL	<14.0 APL
Cardiolipin IgG	1.0 GPL	<10.0 GPL
Cardiolipin IgM	1.5 MPL	<10.0 MPL
ß-2 glycoprotein 1 IgA	2.5 U/mL	<7.0 U/mL
ß-2 glycoprotein 1 IgG	1.6 U/mL	<7.0 U/mL
ß-2 glycoprotein 1 IgM	<2.4 U/mL	<7.0 U/mL
Prothrombin 20210A mutation	Negative (homozygous wild type)	-
Factor V Leiden mutation	Negative (homozygous wild type)	-
Lupus anticoagulant (Hex phosph neut test, DRVVT)	Negative, DRVVT <44 sec	DRVVT <44 sec
Factor V activity	86%	62-140%
JAK2 V617F mutation	Negative (<0.1%)	<0.1%
JAK2 exon 12 mutation	Not detected	-

## Discussion

This case illustrates how CVST can present as a stroke mimic during an acute neurologic evaluation. The abrupt onset of unilateral weakness appropriately triggered a stroke alert, yet venous imaging ultimately revealed the underlying diagnosis. In retrospect, the most important clinical clue was the subacute history of persistent headache preceding the focal deficit. When headache precedes neurologic deficits and arterial imaging is unrevealing, CVST should remain a diagnostic consideration.

The differential diagnosis at presentation was broad. Arterial ischemic stroke was prioritized due to the acute focal deficit. However, normal noncontrast CT findings and the absence of perfusion mismatch reduced concern for an evolving large arterial infarction. Subarachnoid hemorrhage and infectious etiologies were less consistent with the clinical course and imaging findings. Although cervical musculoskeletal strain was initially considered as a cause of his neck pain, it could not account for the subsequent development of extensive dural venous sinus thrombosis or focal neurologic deficits. CVST accounted for both the headache pattern and transient focal deficits. Headache is the most common presenting symptom in CVST and may occur early, while focal deficits and seizures are more common with extensive disease [1-3,7,8].

A second learning point is that extensive clot burden may occur even when a thorough hypercoagulable evaluation is unrevealing. CVST is associated with numerous inherited and acquired prothrombotic conditions, yet a subset of patients remains without an identifiable cause after evaluation [2-6]. This patient had features that raised concern for hypercoagulability, including a remote pulmonary embolism, family history of venous thromboembolism, and elevated hemoglobin and platelet count at presentation. Despite this, comprehensive testing was negative. The anatomic extent was also notable, with thrombosis extending from the superior sagittal sinus into the transverse and sigmoid sinuses and the distal internal jugular vein. Extension involving the jugular vein has been reported, though the directionality and timing may be difficult to establish in individual cases [12].

Despite an extensive evaluation, no definitive provoking factor for this patient’s CVST was identified. Testing for inherited and acquired thrombophilias, including myeloproliferative neoplasms and antiphospholipid antibodies, was unrevealing. There was no clinical evidence of dehydration, active malignancy, or systemic inflammatory disease. Although a remote tick bite was noted in the history, the infectious evaluation was negative, and there was no clinical or laboratory evidence to suggest a causal relationship. As described in the prior series, a subset of CVST cases remains idiopathic despite comprehensive evaluation, underscoring the multifactorial nature of venous thrombosis.

Prognosis in CVST is variable. Data from ISCVT show that most patients recover functional independence, though a meaningful minority experience severe disability or death [5]. Poor outcomes have been associated with depressed consciousness, focal neurologic deficits, and deep venous system involvement [9,10]. Intracranial hemorrhage at presentation is another marker of more severe disease [11]. Despite extensive multisinus involvement in this case, the absence of deep venous thrombosis, hemorrhagic venous infarction, and clinical deterioration supported continued anticoagulation without escalation.

Anticoagulation remains the foundation of CVST management [1-3]. The use of intravenous heparin followed by transition to a direct oral anticoagulant (DOAC) aligns with contemporary practice and emerging evidence supporting DOACs for long-term therapy in selected patients; however, randomized data remain limited, and careful patient selection is important when considering DOACs for long-term management [1,3]. Endovascular interventions are generally reserved for patients who worsen or fail to improve with anticoagulation, as available series emphasize hemorrhagic risk and careful patient selection [15,17]. Risk stratification tools such as PRACT-CVT may help identify patients at higher risk for deterioration who could benefit from early consideration of escalation [16]. In this case, the absence of deep venous system involvement, intracranial hemorrhage, and clinical deterioration would place the patient in a lower-risk category, supporting continued anticoagulation without escalation [16]. This case reinforces that clot burden alone should not drive decisions regarding invasive therapy.

Follow-up after CVST should include monitoring for recurrence and delayed complications. Dural arteriovenous fistulas may coexist with CVST or develop later and should be considered in patients with persistent or recurrent symptoms [13,14]. Patients should also be counseled that headache or fatigue may persist despite good functional recovery, which can help set expectations during recovery [2].

## Conclusions

Although conclusions are drawn from a single case, CVST can closely resemble arterial stroke and should be considered in patients presenting with focal neurologic deficits preceded by persistent headache, particularly when initial arterial imaging is unrevealing. This case shows how early venous imaging in stroke evaluations is important when radiologic findings are not fully explained by the clinical presentation. It also demonstrates that extensive multisinus thrombosis may occur despite a comprehensive and unrevealing thrombophilia evaluation. Anticoagulation remains the cornerstone of treatment, and escalation to endovascular therapy should be based on clinical trajectory and anatomic risk features rather than clot burden in isolation.
